# Understanding the Impact of Equitable Collaborations between Science Institutions and Community-Based Organizations: Improving Science through Community-Led Research

**DOI:** 10.1093/biosci/biac001

**Published:** 2022-03-22

**Authors:** The ICBOs and Allies Workgroup, María Cecilia Alvarez Ricalde, John Annoni, Rick Bonney, J Marcelo Bonta, Patricia B Campbell, Mateo Luna Castelli, Makeda Cheatom, Catherine Crum, Juan Flores Valadez, Yao Augustine Foli, José González, José Miguel Hernández Hurtado, Sister Sharon Horace, Karen Kitchen, Marilú López Fretts, Brigid Lucey, Pepe Marcos-Iga, Karen Purcell, Berenice Rodriguez, Tanya Schuh, Phyllis Edwards Turner, Fanny Villarreal, Bobby Wilson

**Affiliations:** Green Jay Bird Conservancy, Cancún, Quintana Roo, Mexico; Camp Compass Academy, Allentown, Pennsylvania, United States; Cornell Lab of Ornithology, Cornell University, Ithaca, New York, United States; J.E.D.I. Heart, Portland, Oregon, United States; Campbell-Kibler Associates, Groton, Massachusetts, United States; Cornell Lab of Ornithology, Cornell University, Ithaca, New York, United States; WorldBeat Cultural Center, San Diego, California, United States; MSW, located Washington, DC, United States; Green Jay Bird Conservancy, Cancún, Quintana Roo, Mexico; Ndor Eco Village, Hohoe, Ghana; The Avarna Group, Bozeman, Montana, United States; La Joven Guardia del Teatro Latino, Syracuse, New York, United States; located in Harlingen, Texas, United States; Cornell Lab of Ornithology, Cornell University, Ithaca, New York, United States; Cornell Lab of Ornithology, Cornell University, Ithaca, New York, United States; Cornell Lab of Ornithology, Cornell University, Ithaca, New York, United States; Cornell Lab of Ornithology, Cornell University, Ithaca, New York, United States; WorldBeat Cultural Center, San Diego, California, United States; Metro Atlanta Urban Farm, College Park, Georgia, United States

**Keywords:** equity, diversity, justice, inclusion, community

## Abstract

To advance justice, equity, diversity, and inclusion in science, we must first understand and improve the dominant-culture frameworks that impede progress and, second, we must intentionally create more equitable models. The present authors call ourselves the ICBOs and Allies Workgroup (ICBOs stands for independent community-based organizations), and we represent communities historically excluded from the sciences. Together with institutional allies and advisors, we began our research because we wanted our voices to be heard, and we hoped to bring a different perspective to doing science with and not on communities. We created a community framework to guide our research and we led all aspects of our work, from creating research protocols to analyzing and interpreting the data to disseminating the results. We share our research framework, methods, and results so that science institutions can better understand how to intentionally create more equitable research partnerships with our communities.

“People say success begets success, but I think feeding inequity begets more inequity. The key drivers are understanding how you are gonna leverage that power to close the equity gap.”

(All quotes in the article are from our community research responses.)

Despite strong efforts by many scientific institutions, such as universities and large museums, and their funders to increase diversity and equity in the sciences, little meaningful progress has been made (NSF 2017, Pew Research Center 2018, Forrester 2020). In many culturally diverse and minoritized communities, members experience the sciences not through institutions external to their community (such as universities or large museums) but via community-based organizations (CBOs), such as churches, advocacy centers, or community centers. These community members are more likely to engage with CBOs that they know and trust than with large academic or informal science education institutions they do not know, even if those institutions are located in their communities. However, CBOs rarely receive funding to develop scientific research and outreach programs in their communities because they are typically perceived by funders as lacking fiscal stability to support large awards or as having insufficient academic expertise. Rather, the scientific enterprise is systemically structured such that large scientific institutions receive the majority of available funding and set programming, research, outreach, and engagement priorities for communities and their members. Dominant-culture institutions are, essentially, gatekeepers to the sciences.

To help understand why increasing justice, equity, diversity, and inclusion (JEDI) in the sciences remains mostly stagnant, consider that many of the frameworks and approaches used to study and improve JEDI have been informed largely by dominant-culture worldviews and typically maintained by scientific institutions (Bonilla-Silva and Baiocchi 2001, Irwin 2006, Wynne 2006, Bonilla-Silva 2009, Harper 2012, Medin and Bang 2014, Lyons 2017). As a consequence, little research has been focused on issues or concerns that readily emerge from community-based perspectives. Therefore, although scientific institutions often have a desire to support JEDI conceptually, in practice, they frequently reward behaviors and research approaches that do not actually enact or promote JEDI and may actively create barriers to increasing it. To overcome this problem, we must examine power, privilege, and race within the scientific enterprise and deconstruct the process of science itself by confronting a history of ongoing colonialism and white supremacy. There is an urgent need for scientific institutions to focus on antiracism, humility, and reciprocity and to attend to the human side of the sciences by learning “how to ethically engage with Indigenous peoples” (Littlechild et al. 2021) and other communities historically excluded from the field.

To better understand the key touchpoints that influence equitable collaborations between STEM institutions and CBOs, in the present study, we set out to better understand the following question: “What are the factors that influence how STEM institutions collaborate equitably with CBOs to implement scientific research and programming in communities historically excluded from the sciences?” In addition, we wanted to explore how community values play out in collaborations, research, and STEM program implementation.

“Just because you understand equity and are committed to equity doesn't mean you're immune to falling into the traps of dominant culture or inequity.”

A review of the literature suggests that when scientific institutions partner with CBOs to conduct research in underserved communities, they should be respectful of the communities involved, increase communication, share results, value traditional ways of knowing, and consider who benefits from the research (Davis and Reid 1999, Koster et al. 2012). The recommendations for achieving more equitable collaborations between scientific institutions and CBOs have been focused on communication, philosophy, purpose, commitment, underfunding, strengthening the pipeline, and creating a welcoming environment (Mattessich et al. 2001, Cameron and Lart 2003, Tsui 2007, Porticella et al. 2013, McCarthy and Herring 2015). However, these recommendations typically encourage vague and passive external solutions that seldom address scientific institutional culture; change personal behaviors; or address white supremacy culture, colonialism, institutional racism, and inequitable collaborations with minoritized communities in significant ways. For instance, many scientific institutions assert that the pool of competitive candidates who are diverse in the STEM pipeline is inadequate or that their “doors are wide open” and that it is “patronizing” to do things differently when working with minoritized communities. In addition, researchers continue to minimize or ignore institutional racism and academic norms that overlook the realities and policy priorities of minoritized communities (Bonilla-Silva and Baiocchi 2001, Bonilla-Silva 2009, Harper 2012).

“What are the things I'm looking to assess?… One is a willingness to have uncomfortable conversations. Another is… the analysis around power and privilege and around equity, because that's gonna be really important, especially if we're at different powers of privilege as we enter this relationship.”

To make meaningful progress toward achieving greater JEDI in the sciences, we must confront inequities, racism, and power dynamics within the scientific enterprise, dominant culture institutions, and academia. Traditionally, broadening and increasing participation in science assumes that science is neutral and provides benefit to everyone by moving knowledge forward. However, science is not neutral or unbiased (Bonilla-Silva and Baiocchi 2001, Minkler et al. 2008a, b, Harper 2012, Koster et al. 2012, Balaz and Morello-Frosch 2013). Neutrality and objectivity in traditional science assume assimilation into the dominant culture's science identity (Carlone and Johnson 2007). And when it may feel necessary for the sciences to fall back on fixed or entrenched traditional scientific practices, it is critically important to reflect on the necessity of confronting social bias, white supremacy thinking, and a history of colonization and oppression (Trisos et al. 2021). Failing to do so “may be used to suppress otherwise valid dissenting positions” and directly conflict with objectivity (Harding 2015, Prescod-Weinstein 2020).

The sciences can provide valuable tools to learn about the world and advance knowledge, but if the research is to occur in communities historically excluded from the sciences, our findings argue that it should include people who have deep knowledge (including lived experience) of the issues of power, privilege, and race throughout the entire research process. Our results indicate a need for a more balanced research approach that explores the value of equitable transfer of expertise from the communities to the sciences and from the sciences to the communities. In the present article, we—leaders, representatives, and allies of CBOs in communities historically excluded from the sciences—share our research on and discussions about how to significantly achieve greater JEDI in scientific research and programming. We describe how we used community-based participatory research (CBPR; Israel et al. 1998), grounded theory (Corbin and Strauss 1990), and critical race theory (Delgado and Stefancic 2017) approaches to first develop a conceptual community framework to better understand equitable collaborations between scientific institutions and CBOs and second to explore barriers and strategies to creating meaningful change.

## The ICBO story

In 2013, the Cornell Lab of Ornithology, the Association of Science-Technology Centers, and the Garibay Group launched a collaborative research project funded by the National Science Foundation called “Examining Contextual Factors that Influence the Implementation of Projects Designed to Improve Cultural Diversity in Informal STEM Programming.” The goal of this implementation research was to better understand factors that influence partnerships between scientific institutions and CBOs when implementing informal science programs in minoritized communities. The research plan was to study interactions among dyads. Five scientific institutions would select CBOs and work together to implement a citizen science project in five cities across the United States, and project researchers would determine barriers to and successes resulting from the partnerships. In addition to the scientific institutions and CBOs, the study included the present authors—a group of 13 community advisors, the ICBOs (for independent community-based organizations), who each represented one or more historically marginalized communities. The role of the ICBOs was to reflect on and inform the implementation research focused on the dyads, and most of the present authors represent one of those ICBOs.

By the winter of 2016, the ICBOs had become frustrated with the research framework and related frameworks used by dominant-culture institutions to study minoritized communities, because these frameworks did not explore power or race, and they did not prioritize worldviews from our communities in research. We realized that dominant-culture research that is focused on understanding diverse communities often yields inaccurate results because the research questions, data collection, and interpretation of results all lack the unfiltered worldviews of our communities (see Minkler 2005). Therefore, we decided to lead an autonomous strand of research shaped by our points of view and our community experiences. To address our cultural norms, priorities, neighborhood characteristics, unspoken considerations, tensions, and fears, we developed a study to better understand how to honor the priorities of our communities (see Wallerstein 2006, Jones and Wells 2007). We conducted this research mostly on our own time, because, in the grant, the ICBOs received small stipends as advisors but no funding to conduct original research.

Our research was focused on better understanding collaborations between CBOs and scientific institutions and included a cocreated, qualitative survey or interview protocol that included 30 questions (see appendix A in the supplemental material). The study participants were members of diverse, marginalized, low-income, and minoritized communities located in College Park, Georgia; Westside and Southside Syracuse, New York; Germantown, Pennsylvania; Washington, DC; San Diego, California; Allentown, Pennsylvania; Portland, Oregon; Saint Paul, Minnesota in the United States; and Cancún, México; the Lake Volta region of West Ghana; and San Juan, Puerto Rico; among others. The communities included African Americans, Latinos, Indigenous Americans, Africans, Muslims, migrants, and people experiencing homelessness. They included representatives from many types of organizations and niches, including hunting or fishing, arts, health, music, rehabilitation, religious, advocacy, theatre, education, homeless support, food deserts, and urban farming. The study participants included 20 women and 11 men, the majority in their mid- or late careers.

The participants also included diverse staff from the Cornell Lab of Ornithology, a well-known science and conservation institution, which is the only dominant-culture institution present in the ICBO collaboration. The partnership between the Cornell Lab and the community researchers hasn't been easy and has required navigating power dynamics and roles over the entire course of the research. From the beginning, the lab and the ICBOs needed to build trust and to transparently understand how power, privilege, and race affect systems and collaborations. The participants also needed to determine the lab's role in the research itself. We continue to navigate historic tensions and divisions between traditional research and community-based research at the time of this writing. For the most part, the lab has served as a research advisor for the ICBOs, most with no background in social science research. In addition, the lab has provided the platform for the research (Cornell Qualtrics) and serves as a central repository for project data. One of the keys to success has been for the collaboration to be led authentically by the ICBOs and for the lab to follow the ICBO lead and to not interfere or attempt to influence collective decisions, values, or rules. Together, we believe that genuine community engagement in science should “represent a challenge to existing assumptions about the nature of scientific expertise” and feel that it is important to redefine a “scientific problem in terms of community interests” (Chari et al. 2017).

### Community-based participatory research, grounded theory, and critical race theory

The ICBOs aspired to work with research theories that would embrace community leadership throughout the research process, would center race in the sciences and in scientific research and uncover structural racism in the scientific enterprise, and would be guided by their communities’ worldviews and realities instead of traditional dominant culture perspectives. In this spirit we chose research theories that most aligned with these principles.

Equitable research builds on community priorities, and we acknowledge and reject a historical dynamic that leaves communities feeling studied and used (Davis and Reid 1999). Instead, we chose CBPR as a research approach that directly and equitably involves those affected by an issue in research and action involving social change (Minkler et al. 2008a, b, Balaz and Morello-Frosch 2013). It involves affected community members, researchers, and community organizations in all aspects of the research process and provides a structured manner with which to understand and solve challenges by combining knowledge with action to achieve social transformation (Wynne 2006). CBPR is a collaborative approach that “elevates community knowledge, challenges traditional power dynamics in the research process, and can directly benefit the communities involved” (Balaz and Morello-Frosch 2013). The CBPR methodology highlights the importance of direct community involvement throughout the research process from the development of the research questions and design of the study to the analysis, interpretation, and dissemination of results (Israel et al. 1998). This methodology allows the community to determine the issues to be studied and to disseminate the results with the purpose of addressing those issues (Israel et al. 1983, Davis and Reid 1999). Communities that have participated in CBPR have “sought to democratize knowledge production in ways that transform research from a top-down, expert-driven process into one of colearning and coproduction” (Israel et al. 1998).

In addition, we used grounded theory (Corbin and Strauss 1990, Breckenridge and Jones 2009) approaches to develop explanatory theories (Breckenridge and Jones 2009, Delgado and Stefancic 2017) about the lack of JEDI in the sciences and to better understand the barriers and strategies used to achieve more equitable collaborations between CBOs and scientific institutions. We believe that explanatory theories and framing should emerge directly from the data, because the data represent our lived experiences and meaning-making (see Breckenridge and Jones 2009). Our goal was to share the experiences and knowledge that we had acquired through our years working directly within our communities. The ICBOs did not want to begin with an a priori list of issues to explore that emerged from literature established by dominant-culture institutions, because that approach would fail to uncover the rich description of what mattered to the community members themselves. Therefore, to guide our research, we used grounded theory to create a community-based, holistic framework of factors that influence JEDI in the sciences and in partnerships.

The ICBOs also understood that objectivity, neutrality, and rationality in research is expected in the sciences, and we balanced this with the reality of our lived experiences. We used critical race theory as an interdisciplinary approach (Delgado and Stefancic 2017) to examine the consequences implied in the idea that racism might be understood as a subjective construct (Bell 1992, Ladson-Billings and Tate 1995, Collins 2015). Racism is a persistent and permanent part of society, and critical race theory helped us better integrate context into our analysis and consider experiential knowledge. Throughout our research journey, we also were guided by the principles of empowerment education (Freire 1970) and constructivism (Patton 1990).

### Phase 1: setting the stage for equitable research

The ICBOs felt it was important to explore issues of power and privilege, race (including institutional racism), and trust and transparency as central components of our research on better understanding the role of collaborations between CBOs and scientific institutions; however, to do the research, we needed to collaborate with an academic research partner (the Cornell Lab of Ornithology, a dominant-culture scientific institution). Because the desire to initiate community-led research stemmed from us and because our partners at the lab seemed to have a genuine desire to understand our concerns and priorities, we were able to establish more equitable power dynamics and avoid some of the tensions often experienced by minoritized communities of feeling “researched” and “excluded” from the academic process (Davis and Reid 1999).

**Figure 1.**
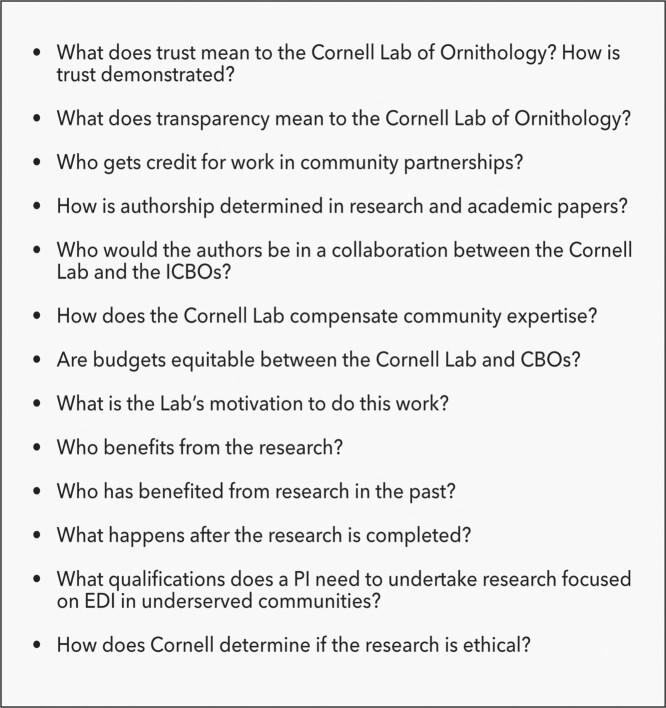
Questions explored before beginning the research.

Although all of the ICBOs had a history of partnering with the lab before joining the project—mostly by engaging in lab-led citizen science projects—we had never collaborated on issues of JEDI. We understood that the lab's motivations for taking on this research, like most large scientific institutions, was connected to a dismal track record in JEDI. However, although in previous collaborations we had an understanding and guarded commitment to working together, we had never developed genuine trust. As we began our new collaboration, the ICBOs wanted to explore the historical track record of the lab in previous collaborations. For instance, we explored who generally gets credit for work in community partnerships at the lab, how authorship is determined, how grant awards are managed, and how community expertise is compensated. We were not surprised to learn that research consistently benefits the lab over community partners (figure 1). We examined budgets in existing and past collaborations and discussed systemic issues such as the university's requirement of over 65% indirect costs in budgets.

Because the ICBOs each represented culturally diverse communities with different priorities, histories, needs, and strengths, there were tensions that needed to be addressed among us. We wanted to understand each other's motivations and the historical relationships among the participating cultures. These tensions became central topics of discussions that would guide our research. For instance, an African American ICBO wanted to explore antiblackness in Perú, one of the countries of origin of another ICBO, whereas others wanted to better understand the consequences of progun attitudes in community programming via hunting. There are marked differences in perspectives, approaches, and worldviews among the ICBOs. Time was needed to develop trust and work through conflicts.

In working with a dominant culture institution with more power, systemic neglect of equity, widespread white fragility, and a history of systemic racism (as is true for most STEM institutions), we also needed to create safe spaces for us to explore the research, power, and collaboration dynamics described in the previous paragraph. During the initial stages of the project the ICBOs met independently—without lab staff—because even though some lab employees working on the project were culturally diverse and aware individuals, their positions at a dominant-culture institution automatically conferred more power and privilege. Meeting independently allowed us to have complete autonomy to explore issues concerning the research and collaboration. We created intentional spaces in which to meet alone during monthly calls and in-person meetings. These spaces generated a growing sense of trust and respect, ensured that we could drive the research in the direction that we felt necessary, and prevented us from inadvertently slipping into framing from the dominant culture that did not reflect our lived experiences.

**Figure 2.**
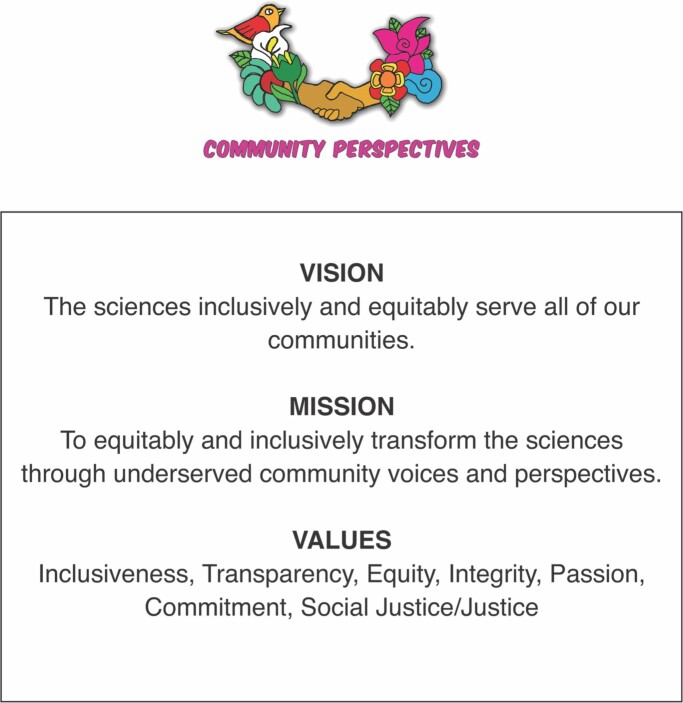
ICBO mission, vision, and values.

**Figure 3.**
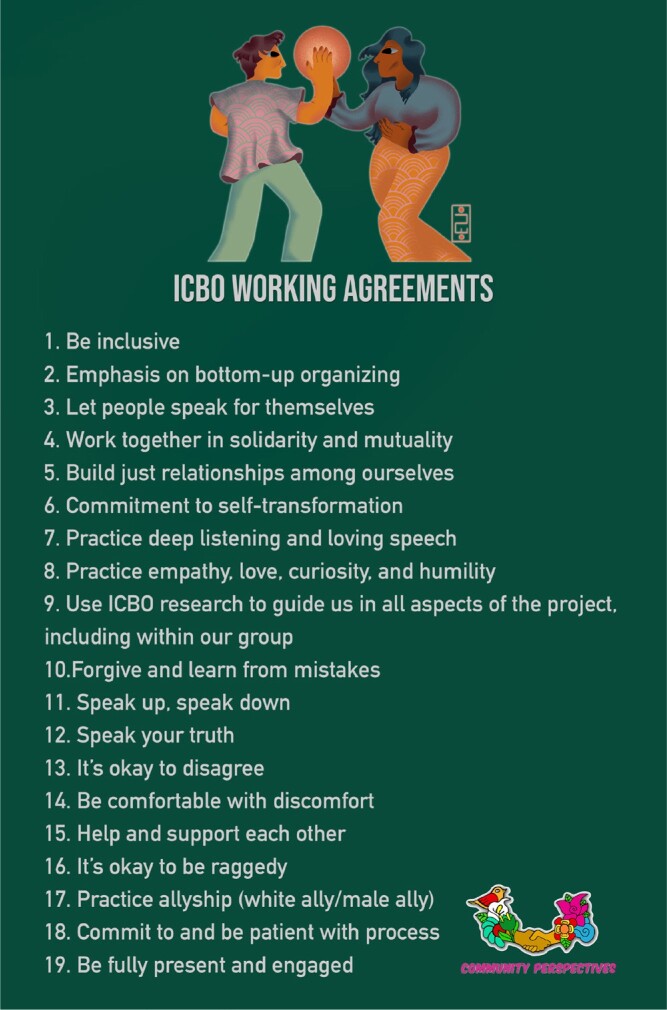
ICBO working agreements based on the Jemez Principles for Democratic Organizing. Illustration: community researcher Jaliliah Williams.

As ICBOs, we felt it was important to carry out our research and dissemination of results embedded in democratically agreed-on values, transparency, and clarity. We established a vision, mission, and prioritized our values (figure 2). Our guiding agreements were developed at an in-person meeting at one of the ICBO partner sites. We developed ICBO working agreements (figure 3), based on the Jemez Principles for Democratic Organizing (Solis and Union 1997), to guide our process and decision-making. We read our working agreements in English and Spanish before our meetings and often referred to them when faced with challenges. Over the years, we have changed and adapted our agreements as our team has grown and developed.

**Figure 4.**
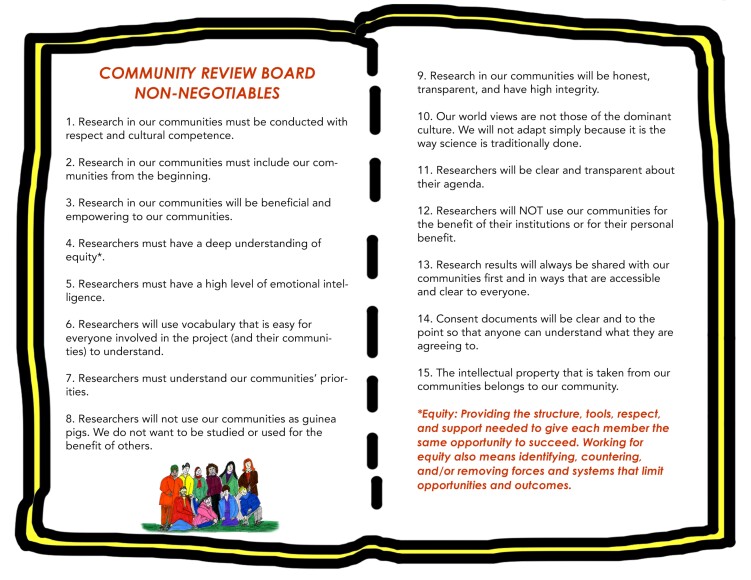
Community review board of nonnegotiables to guide research and evaluation in our communities.

The ICBOs also created nonnegotiables for doing research and evaluation in our communities (figure 4). For most of the ICBOs, the IRB process was new, and although it appears to effectively safeguard institutions from legal challenges, the system does not seem to consider the ethical challenges of balancing power, benefit, and oppression in scientific research. The IRB process does not effectively center our communities’ perspectives, realities, or priorities, and does not thoughtfully support community-led research approaches. Authors such as Tuck and Guishard (2013) do an excellent job in documenting issues stemming from institutional IRB processes centered on safeguarding individual rights and autonomy and protecting institutions from legal challenges. To balance the IRB system, we decided to create a document that centers on community priorities and nonnegotiables focused on social justice and equity. We cocreated our living Community Review Board of Non-Negotiables to Guide Research and Evaluation in our Communities to help guide our team and others in conducting research in minoritized communities. We did this at an in-person meeting followed up with emails, phone calls, and texts. This is a living document that we update as we see necessary. Our nonnegotiables guide our research process. We are not the first community to create a document outlining criteria and processes parallel to the IRB process. We recommend that readers look at a variety of examples, including The West End Revitalization Association's community-owned and -managed research model (Heaney et al. 2007).

At the beginning of our research journey, we agreed that we wanted to compile (and continuously add to) a CBO strengths and resources inventory. We wanted to lead with a clear understanding of the strengths that our communities already have in the research and collaboration space. Instead of thinking of our communities as places that are in deficit or in need of fixing, the ICBOs felt it was important to begin from a place of strength and priorities. The strengths in the assets inventory include the following: We have CBOs with staff that live in the community and reflect community values. Our CBOs have gained the trust of our community (in particular with undocumented immigrants). And we have CBOs with staff that speak the languages spoken in our community. The inventory became an important document that we referred to often in our work, because it helped to guide and frame discussions. Created through self-reflection, the inventory is a living document that will continue to grow and change as we move forward. Each ICBO reflected on its organization's and community's strengths and assets. Our partners shared strengths via phone meetings and an email survey. The inventory helped to provide context for identifying our research questions and protocol.

### Phase 2: developing a community research framework

**Figure 5.**
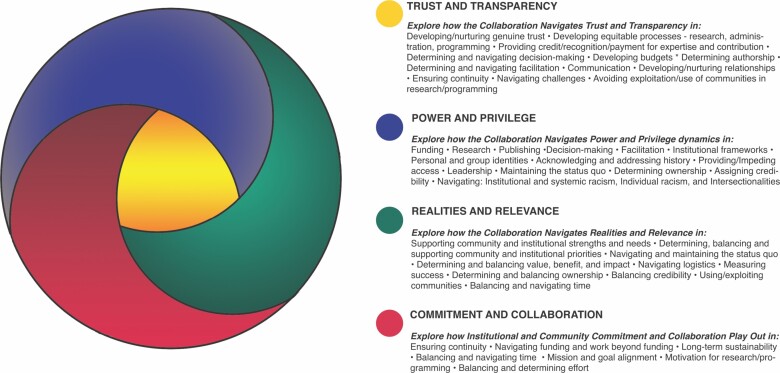
Initial ICBO cocreated community framework. Illustration: Marilú López Fretts.

As the ICBOs set the stage to conduct our research—guided by our mission, vision, values, working agreements and nonnegotiables—we explored the value of established research frameworks and the tensions of using frameworks grounded in dominant culture literature and worldviews. Based on our meeting notes, cocreated mission, vision, values, agreements and nonnegotiables, we identified four major categories, which became the basis for the ICBO framework that guided our research (figure 5): power and privilege, trust and transparency, realities and relevance, and commitment and collaboration. Although our framework initially provided structure and direction to our research, it also evolved iteratively as we analyzed and better understood our findings. Our first iteration of the community framework centered on trust and transparency and can be seen below. We discuss this process and share our final community framework in the results section of this article.

The community framework has been at the center of our work, both in the development and evolution of our working agreements, nonnegotiables, and ways of working together and in the development of the research protocols, community coding, analysis, and dissemination. For instance, one of the ICBOs used our community framework to structure a sermon he delivered focused on our research results. Our framework has helped our team shape the development of equitable processes and navigate tensions both within the ICBOs and in our work with the Cornell Lab around issues of race, ethnicity, and gender.

### Phase 3: developing the protocols for the study

Our goal was to conduct a study parallel to the one conducted by the original research team. The research questions posed by the institution were these: What are the contextual factors that influence how a science program is implemented in different communities? What are the values that play out both in the collaborations and the program implementation? The ICBOs framed these questions within the context of power, privilege, trust, transparency, and race and racism. We asked, “What are the factors that influence how STEM institutions collaborate equitably with CBOs to implement scientific research and programming in communities historically excluded from the sciences? What are the community values and how do they play out in collaborations and program implementation?” Guided by these questions, the ICBOs centered community perspectives by using the community framework to develop research protocols and be guided directly by communities historically excluded from the sciences. Most important, we wanted the research to be and to feel inclusive and did not want research participants to feel as though they were being studied.

To ensure that the data collection instruments and protocols were community centered, the ICBOs were guided by our nonnegotiables and further agreed to develop protocols that highlighted the following criteria: data collection instruments that work for the practical realities of the CBOs and community members who would participate in the research (i.e., time or scheduling limitations, trust and safety with science and institutions), data collection instruments in the language that is most applicable for our communities (English and Spanish), data collection instruments and protocols that are appropriate for different literacy levels of participants (visual or spoken), and incentives that are appropriate for community participants taking part in the research considering the expected time commitment.

Staff from the Cornell Lab, together with some ICBOs with experience in survey design, held an informal training in which we discussed the principles of developing survey questions. We touched on open versus closed questions, question order, developing questions that would not lead participants, ensuring that each question asked only one question, question clarity, and more. Following our community-centered research agreements, each of the ICBOs suggested three questions under each of our community framework categories. We collected these questions using the Survey Monkey platform. Then, on group conference calls, we collectively consolidated and refined the questions to reach agreement about the intended meaning of each. This process resulted in thirty open-ended questions. We decided that we did not want to use questions that led to closed-ended or multiple-choice answers because we did not want to limit the capacity for participants to share their expertise. Because of the number, depth, and length of the questions, this meant that the questions would be best suited to an interview protocol. It would also be important for the interviews to be conducted by the ICBOs themselves. It would not be appropriate for staff (even staff of color) from a dominant-culture institution such as the Cornell Lab to lead the interviews, even if the questions were created by the ICBOs, because that process could cause perceived and real power dynamics that might bias the research.

Ideally the ICBOs would work in pairs to interview each other, in addition to at least one additional invited community participant. However, although it might have been best to use an interview protocol to collect the answers to our questions, during discussions we determined that it would be extremely challenging for us to dedicate the time to training on interview protocols, conducting at least one or two interviews, and being interviewed ourselves. Many members of the ICBOs work more than one job in our communities and have community obligations that would not allow us to dedicate so much time to this work. Therefore, we decided to use a qualitative written survey protocol instead of conducting interviews, meaning that we would give people enough time to answer the 30 questions in written form. We also ensured that the participants would receive an incentive that would appropriately compensate them for their time. The survey was created in Cornell Qualtrics, and the participants were instructed to answer at their own pace. Furthermore, the participants were asked in the email invitation to write their answers as if they were writing a blog or diary entry. The survey took approximately 2 hours to complete. Although this length is unusual in survey research and might lead to low participation rates, we understood that it was the best we could do in light of our circumstances.

The survey was pilot tested in January 2016 with four participants to see if the protocols worked. The results were varied; some of the respondents struggled to complete the survey, whereas others appreciated having time and space to write their answers. From the pilot, we determined that for some participants it was easiest to share their thoughts verbally, whereas others preferred to do so in written form. We decided that it would be best to give survey participants a choice to take the survey online, be interviewed by ICBOs, or do a combination of both approaches. Three of the ICBOs agreed to conduct the interviews for those who preferred them and to follow-up with participants who had left questions blank or whose answers were unclear in the survey.

A detailed interview protocol guide was developed by Lab of Ornithology staff in collaboration with the interviewers, and training was provided on best practices for interviewing. The interview guide or training helped remind the interviewers to request permission to audiotape the interviews, state the purpose of the interview, explain what would happen with the data, and address confidentiality concerns. It also included a script (approved by Cornell's IRB and all the ICBOs in January 2016) to read before the interview to acquire informed consent. Finally, it helped interviewers identify and avoid verbal and nonverbal behaviors that might unintentionally lead interviewees.

### Recruiting participants and data collection

We used a purposive sampling strategy to recruit additional CBOs to participate in the study. Each ICBO member invited a community leader by verbally explaining the nature of the study to leaders from our communities we felt would contribute to the study through their knowledge and understanding of equity. Most, but not all, of the potential participants were from the same ICBO communities. The ICBOs invited community participants who met the following criteria: They lived and worked in a marginalized community, they were willing to spend 2 hours completing the ICBO survey, and they had collaborated with dominant-culture institutions. We also attempted to recruit community leaders who were varied in the type of community they represented (African American, Indigenous American, Latinx) and the type of CBO they represented (religious, community advocacy or organizing, music, the arts, hunting and fishing). The survey participants were offered a $100 gift card for their participation because of the length, depth, and nature of the survey. We sent out 44 requests for participation in the survey and had a 70% response rate. Some of the community participants declined to participate because of the length of the study.

Thirty-one individuals participated in the research, 15 ICBOs and 16 others. Four individuals preferred interviews, and two survey follow-up discussions were completed. The survey was launched in February 2016 and took the participants 2–6 hours to complete. Most of the participants took over a week to submit the survey. The interviews began in March of 2016 and took approximately 2 hours to complete, with most interviews conducted in two 1-hour sittings. The interviews and follow-up discussions were audiotaped, transcribed independently by Verbal Ink, and checked for accuracy.

### Data analysis

Two staff at the Cornell Lab of Ornithology, two Cornell undergraduate students, and four ICBO members participated in in-person data analysis meetings. Three additional ICBOs participated remotely. The ICBOs also met monthly (some months, weekly) and had two in-person meetings to discuss emerging findings.

All members of the data analysis team were trained by allies and ICBOs with relevant expertise before beginning the analysis, and training continued iteratively throughout the coding process as questions arose. Three individuals were trained via Zoom or conference call. The training sessions generated discussions about the function of codes, coding, different levels of codes and subcodes, and analytic memo writing, as well as the types of coding methods used. All of the team members were encouraged to use in vivo codes as much as possible or to use words or short phrases taken directly from the data to generate codes (Manning 2017). In vivo coding is a useful tool to highlight the unfiltered voices of our communities. Training also covered how to develop themes, categories, and, eventually, theories from the codes and recoding and recategorizing. Finally, all of the members of the team discussed and decided how they would find consensus on the codes, as is described below.

Our approach to community coding was inductive; the analysis did not include any predetermined codes. Individuals involved directly in the analysis met via day retreats involving picnics, whiteboards, colored markers, and chart paper. The interview transcripts and surveys were separated by questions and anonymized. The team always coded as a group, first reading responses individually and then coding in community. The codes were generated directly from the data (see table 1: Sample codes from the ICBO Code Book and ICBO Code Book, appendix B, in the supplemental material) and were centered on our community framework. Content analysis across the framework categories was used to identify themes across the codes. Each person had a booklet of transcripts and surveys divided by question, emerging code book (see the ICBO Code Book, appendix B, in the supplemental material) and thin colored markers with which to code.

**Table 1.** Sample codes from the ICBO Code Book referred to in the text.

Code	Definition
Trickle-down engagement	When institutions put the majority of their funding, staff, and power into programming for dominant culture audiences and expect that it will “trickle down” to the community. When scientific institutions do just enough outreach to obtain funding.
Guinea pigs	Note when respondents mention that community members feel like they are being used in research collaborations. When institutions or more resourced institutions are “using” underserved communities to obtain grants, do research, and check off diversity and inclusion requirements. Note if there is follow-through or sharing research results, and long-term commitment.
Tightrope approach	When ISEs hire one or two people who reflect or represent their communities to do outreach in historically underrepresented communities and these individuals have no decision-making power, may be short-term hires, and may be inexperienced in equity, diversity, and inclusion. Note if the hires have the ability to set the scope of their work or if the ISEs set a narrow scope for the work and have a predetermined approach set by the majority culture (the tightrope).
Third best man	When ISEs send someone, who has no experience in the community, no authority within the institution, and no decision-making power to represent their institution at key planning and negotiation meetings.
I know what you need	Savior syndrome. When top-down programming is implemented in the community even when it is not relevant, wanted, or effective. Note if institutions believe their expertise and resources are best.
Walking on eggshells	When respondents note that CBOs cannot be honest with institutional partners when they see racism, inequity, injustice, or institutional racism. CBOs feel like they are “walking on eggshells,” because addressing inequity might harm their organization or community, prevent them from getting funding, or they may be excluded from a collaboration.
Robin Hood approach	When CBOs knowingly continue engaging with collaborations and partnerships that are untrustworthy, inequitable, frustrating, or lack transparency in order to obtain funds and opportunities that they feel can be channeled directly to their communities.
Know your worth	When CBOs understand their power and worth and communicate it clearly when partnering with ISEs.

We analyzed and hand-coded the answers to each question and then shared all the codes generated for that question on the whiteboard. We then discussed the codes, found consensus on the codes and themes, and grouped and then regrouped the codes into larger categories. Interrater reliability was included in the consensus process. Our coding team did not rely on traditional interrater reliability testing to ensure alignment on the codes. Instead, we came to consensus as we coded while intentionally acknowledging and balancing power and privilege dynamics among the coders. We felt that intercoder reliability testing might create a more intimidating and less inclusive environment. Instead, we aimed to foster a welcoming, nonthreatening, collaborative coding process by openly acknowledging power differentials and inviting difference. Our collective goal was to create a coding process that would lead to results that better represented diverse perspectives through trust building and transparent conversation. Our coding group relied on real-time open discussion to gauge the ways in which coders understood and applied codes and relied on full consensus to finalize the definition and application of codes. Naming codes became important conversations that often required discussion with the larger group. Critical to this work was navigating power and privilege dynamics within our coding team. We understood that lab staff and students might unintentionally become coding authorities because they had prior expertise in social science research. We openly discussed and rejected models of research that rely on authority and power and embraced the value of community expertise. We cocreated working spaces in which questioning biases and codes was the norm and differences in opinions were welcomed and celebrated. During this phase, we expanded and improved the initial set of codes to be more precise and to better reflect the common components that existed across the full data set. New codes that emerged in later questions required that we iteratively recode the previous questions with the new codes. In this way, we created a comprehensive coding dictionary. Disagreements on codes were settled by comparing the codes generated by each coder on a whiteboard, discussing the differences and figuring out why they occurred, and learning from the differences. Any codes for which we could not find consensus were set aside on a separate whiteboard and discussed throughout the remaining analysis until consensus could be achieved.

Finally, Cornell Lab staff uploaded all of the data and coding to an electronic format, using NVivo 11 (QSR International). We identified patterns inductively by analyzing codes associated with each question in our coding retreats, through phone conversations, and in-person meetings. Examination of the response patterns across the entire data set ultimately led to the emergence and identification of larger themes and explanatory theories.

## Challenges and tools

Our results should be looked at on both systemic and individual levels. Please see table 1: Sample codes from ICBO Code Book and the appendix B, in the supplemental material. We coded our qualitative survey and interview responses to highlight the unfiltered community voices, shown in parentheses.

Our findings point to a system that provides CBOs with no direct access to funding streams, research opportunities, or networks, making it nearly impossible for our community voices to be heard directly in the sciences. The system creates a funnel that makes CBOs dependent on dominant-culture institutions for funds and representation in science.

“A larger institution has power… so it's gonna receive a large community engagement grant because a funder may see they have the capacity or really the power to deliver on that. But then, they in turn will engage in trickle-down community engagement, and that's just perpetuating the inequity.”

**Figure 6.**
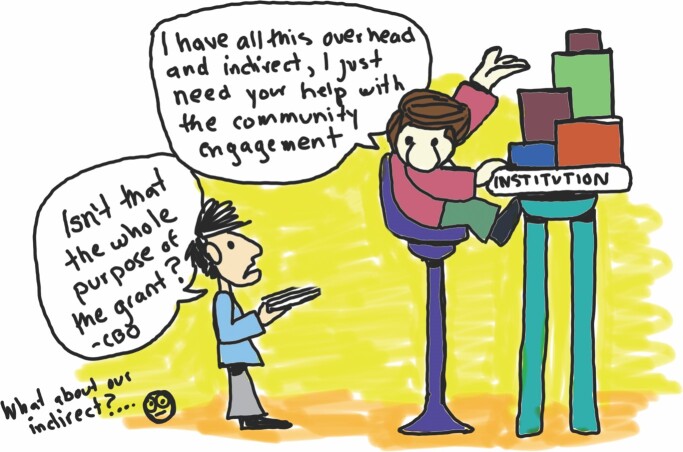
Trickle-down engagement. Illustration: community researcher and ICBO member José González.

Most CBOs in our study believe that scientific institutions primarily place their funding and staff into programming for dominant-culture audiences and expect that it will “trickle down” (trickle-down engagement; see figure 6) and ultimately benefit minoritized communities.

CBOs also feel that exclusion is common both on a systemic level particularly with funding and on an individual level. Examples of exclusion include using technical language, choosing meeting venues that are difficult to access or feel exclusionary, not showing up to or inviting CBOs to key meetings, requiring advanced degrees or valuing degrees more than experience, not paying attention at meetings, and disseminating results via channels that exclude nonacademics.

“At first, you might spend years doing it. Dress like them, speak their lingo, network, go to meetings in hopes that your organization will be accepted. You hope you will be accepted if they know your vision. Eventually, you realize that they are actually racist and ignorant of the experience of people of color. And then you realize they partly don't care because they are elitists, and then you realize you don't want to be part of their club anyway. You then realize that you were never a part of the “club” and understand why you missed out on meetings and other activities.”

Our findings point to a strong theme of exhaustion with a system that leaves our communities feeling studied and used without clear benefits (Guinea pigs). The sciences do not easily allow us, the affected communities, to ask the questions based on our priorities, analyze the findings, interpret the results, and disseminate the results in ways that are accessible to our communities. Often, efforts to do so are dismissed, labeled as biased or simply ignored.

“I felt more like [we] were guinea pigs and there was no real support built in.… And again, I understand it's for research's sake, but these are real people, and these are real relationships… like, there are real kinds of implications, I guess, and consequences.”

Changing a system that has oppressed our communities for so long and has become cemented into the framework of scientific institutions has many challenges. Our findings suggest that institutional racism may be invisible to scientific institutions. We examined our findings within the context of critical race theory (Delgado and Stefancic 2017) and believe that a critical understanding of societal constructions of race is essential to our analysis. One of the underlying aspects of critical race theory is the idea that racism is so ingrained and normalized in our society that we can no longer see it (Bell 1992, Delgado and Stefancic 2017). But in our analysis of critical race theory, we find there has been a gap in the literature focused specifically on the scientific enterprise. We suspect that the lack of careful examination of color-blindness in the scientific enterprise may expose the continued assumption that science is “a place that is already color-blind because it rid itself of its racist past” and is, perhaps, now immune to racism because “good science is supposed to be objective and politically neutral” (Collins 2015). Consequently, trying to fix or change a system that prides itself on being objective, neutral, and no longer afflicted by racist ideology, is extremely difficult without first acknowledging that a problem exists. We hope that the recent work of scholars such as Prescod-Weinstein (2021) addressing racism in science directly, from a creative, strengths-based perspective, continues to receive serious attention and can help move the field forward.

“In the end, let's say you have programs for youth and all you see is white youth coming to these programs. That's institutional racism “cause you didn't do anything intentional to support and provide the benefits for youth of color. It's not the intent; it's the impact. It's the outcome where institutional racism comes in. You can have the intent, on the front end, of “I don't wanna be racist,” but you provide more benefits to white people than people of color. That's what institutional racism is. That's how it plays out within our system.”

The research participants pointed out that some practices which scientific institutions have put in place to “solve” JEDI barriers might do more harm than good. For instance, hiring one or two people of color to do “community outreach” without providing reasonable budgets or decision-making power (the tightrope approach), sending institutional representatives without authority to meet with minoritized communities (third best man), implementing top-down approaches meant to “save” or “fix” our communities (I know what you need or the savior syndrome), or obtaining funds to work in communities without talking with them first are common and ineffective practices.

“A science institution can believe, “Oh, I have these services and programs that will benefit these communities, so I'm gonna bring them to you and this is how you will benefit from them.” That's completely the wrong way of doing it!”

**Figure 7.**
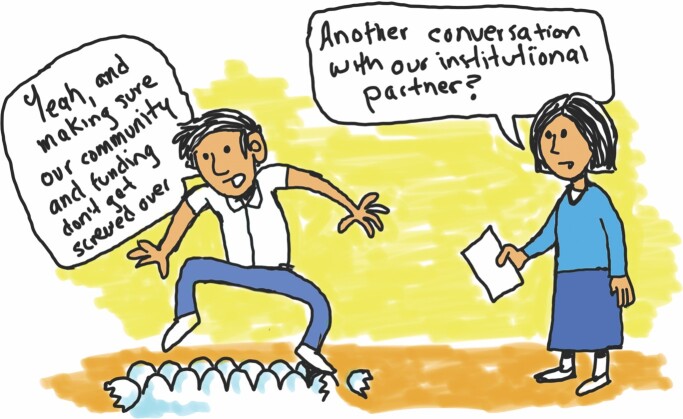
Walking on eggshells. Illustration: community researcher and ICBO member José González.

Our findings also suggest that CBOs are often afraid of repercussions to their communities if they speak up about injustices, racism, and inequities (walking on eggshells; see figure 7). Walking on eggshells is important because it means that CBOs are forced to stay quiet in the face of inequity, whereas scientific institutions have little incentive to change the status quo, perpetuating a system that continues to hurt communities.

“Addressing what may seem to be institutional racism might feel like a confrontation to the bigger institution and if there is a potential that such action may affect the community, I opt to not address it.”

Mistrust runs deep, and most research participants felt frustrated, angry, or powerless in their collaborations with scientific institutions and the scientific enterprise in general. Out of necessity, or because they have become accustomed to the status quo, community leaders sometimes engaged in behaviors that perpetuate the existing system and do not benefit their organizations or communities.

“In my experience, CBOs are so desperate for resources that they give up way too much and reap little benefit in return.”

Some of the study respondents alluded to the Robin Hood approach as a technique that allowed their organizations to stay afloat and their communities to receive needed services. The Robin Hood approach is our in vivo code to describe when CBOs put up with partnerships that are untrustworthy, inequitable, and lack transparency to obtain funds and opportunities that can be channeled directly to their communities. Whether this approach is ultimately effective or detrimental to minoritized communities is unclear.

CBOs did mention approaches that they believed could be effective in creating more equitable collaborations and increasing JEDI. For instance, one approach we coded, as know your worth, identified when CBOs advocate directly for equitable collaborations that value their community's contributions.

“You have something they need. You make it clear that you know what that is. You outline what you are willing to give. You outline what you need in return. You are ready to walk.”

Other approaches mentioned by CBOs included addressing a history of inequity and racism directly and with clarity by acknowledging a history of injustice in the field of science, with a clear plan to overcome past challenges.

“Raising awareness about the history of these disparities and inequities is not just the job of the CBO.”

Research respondents also felt that when scientific institutions and CBOs explicitly state that benefiting minoritized communities is a priority in their missions, they are more likely to have successful collaborations and achieve better impact. However, it is important that institutions live their mission by rewarding and supporting staff and programs that cocreate equitable initiatives and ensure continuity of programming.

“Being part of a mission-driven organization where all members of the organization are encouraged to understand and practice the mission every day and, in every interaction, builds trust and cohesion.”

Spending time in the community (showing up) was a key element named by respondents to demonstrate commitment, increase trust, and gain understanding of the realities of minoritized communities and relevance of initiatives. Connecting on a personal level seems important for establishing motivation, understanding who benefits from the collaboration, working through difficulties, and creating authentic relationships.

“When you see your partners helping out at the community event completely and unrelated to the work you do, but helping the community, you have the extra level of respect and trust and know how committed the partner is.”

### Centering race in our community framework

**Figure 8.**
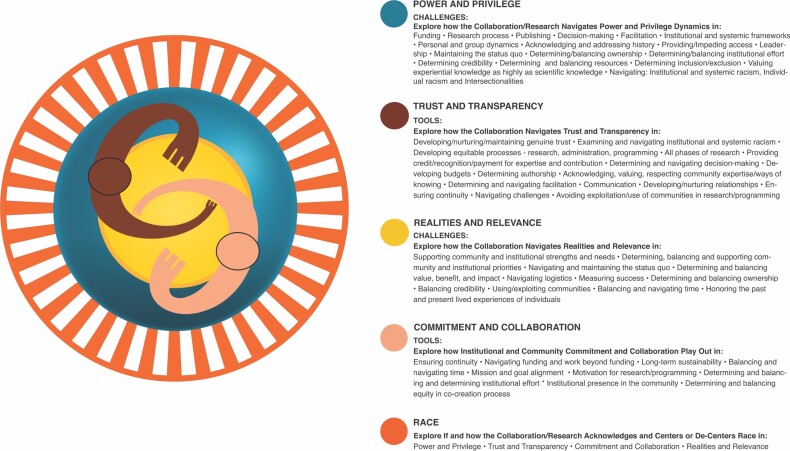
Final version of ICBO Community framework. Illustration: Marilú López Fretts.

Our community framework helped us prioritize our research, and we returned to it often throughout the research journey. Our framework began as a simple guide; the categories of power and privilege, trust and transparency, realities and relevance, and commitment and collaboration originally did not overlap or interplay with one another. We did not see that some categories create barriers, whereas others provide tools with which to navigate those dynamics. With time our framework began to reflect these understandings (figure 8). As our research evolved, our community framework continued to evolve with us. Originally, we didn't include race in the framework at all, but eventually realized that race permeated our research process, questions, and analysis. All our categories were influenced powerfully by race. Race interplayed with tools and barriers and it became evident as our work continued that race had to be considered in all the theme dynamics and interconnections. Without centering race, whiteness, privilege, power, and systemic racism dominate the status quo and collaboration dynamics. Centering race became critical as we used our framework to guide our research and the processes, agreements, nonnegotiables, and decision-making we used to collaborate and implement our work.

## Conclusions

Not all scientific institutions are the same. There is huge diversity in scientific institutions as there is in CBOs and some are much farther along their path to increasing JEDI than others, but, in our work, we found that the balance of power lies within these institutions. Therefore, faced with the challenge and necessity of balancing power and developing trust and transparency in science, our research findings suggest that CBPR is an important approach to studying JEDI because this research technique, if implemented correctly, allows affected communities to ask research questions, work within their values, direct the research in ways that is necessary, interpret the results according to their realities and worldviews, and have ownership of the results. These considerations seem particularly important in view of the strong distrust of scientific institutions and systems in science that uphold the status quo. It is important to note that the ICBOs distrust is not in science itself but in the processes and practices that have been established within scientific research, many of which are informed by a difficult past of extractive and oppressive relationships between scientific institutions and communities. Science can be better and more impactful if it confronts the structural inequities that have historically excluded different ways of knowing, diversity, and difference from institutional research.

“The mistrust [for scientific institutions] runs deep and there will always be seeds of anger and insecurity.”

Although much has been written about using CBPR as a tool to democratize science, and although authentically cocreated CBPR projects led by community members themselves do exist, this model is sparsely represented in the literature (Ottinger 2009, Chari et al. 2017). Typically, CBPR is led by dominant-culture scientific institutions. Even when projects are labeled as cocreated, they are more likely community placed than community based (Minkler et al. 2008a, b). This difference really matters.

“Who is in the room making those decisions? And if it's predominantly white people, you're gonna have decisions that reflect the White-dominant culture.”

Although CBPR shouldn't be used in every research project, in research that directly affects our communities the communities themselves should lead or colead the research, and the impetus for the research should come from us. Furthermore, before any research is undertaken, space must be given to the cocreation of equitable processes, agreements, and nonnegotiables that will guide decision-making, protocols, and dissemination. And, a clear and transparent understanding of the impacts of racism, power, privilege, and racial inequity on the work must be established (Wallerstein et al. 2018).

“Before you go into a partnership, you need to be clear on what your nonnegotiables are. I think that's important to have that discussion from the beginning because, sometimes, partnerships aren't meant to be. For me, it is the commitment to equity. If you don't even have an understanding of what “equity” means, or have a decent understanding of it, or aren't open to learning, then it's not gonna be worth our time and energy to partner.”

CBOs should have direct access to funding streams so that money does not need to be channeled through scientific institutions, and CBOs should have more control to build equitable projects. The infrastructure to support this model does not currently exist within the scientific enterprise. CBOs want access to resources, networks, and channels currently available only to dominant-culture institutions, which will allow them to create or cocreate programming that their communities want and need.

Finally, an important tenet of participatory research identified by Green (2003) is the issue of ownership, or “an explicit agreement between researchers and community participants with respect to ownership and dissemination of the research findings.” A clear agreement, based on ­values, indicating that the “communities will speak for themselves” (Solis and Union 1997) is necessary. Moreover, the ­success of participatory research should be evaluated by how much ownership the communities themselves have over the results. When there are no interpreters or mediating institutions with their own motivations and agendas, we see genuine transparency in the research. As Hicks and colleagues (2012) pointed out, it is important not to operate under the assumption of trust as a given, instead we must “generate trust through our actions; this has been especially critical as we have established memorandums of understanding and expectations that incorporate data ownership, community benefit, and joint publication with case study partners.” Within our ICBO group, ownership of the results has been a strong component of our work. Our collaboration is based on our cocreated working agreements and our community review board of nonnegotiables in research that make it clear that dominant-culture institutions should not speak for us.

“How can we create an inclusive culture… that really honors and respects… the broader narratives and experiences of all people? Because in the end, that's how we're all going to be successful. And if we don't do that, we'll continue to have challenges and not succeed.”

This research suggests that if we are to make meaningful progress in achieving greater equity, justice, and impact in science, we must include the leadership, expertise, and voices of communities historically excluded from the sciences and fundamentally change the scientific enterprise on a systemic and individual level, as M's-it No'kmaq and colleagues (2021) stated.

“It is time for Indigenous voices to take the spotlight. Only after that may authentic re-Indigenized ways of weaving and partnering begin. Indigenous voices need the moment to be all theirs, rather than forced into colonial structures. Insights will then require learning from each other; from the land; from the languages; through reflection and immersion in ecologies, stories, and ceremonies; and revisiting and revitalizing relationships and responsibilities.”

This shift in power, leadership, and narratives can take place only if there is a fundamental change in funding structure so that CBOs are able to receive direct funding to develop research programming and engagement priorities in their communities without gatekeeping from dominant culture institutions. In addition, we believe the adoption of equitable, decolonized participatory research approaches, guided by meaningful cocreated processes that center race, balance power and privilege, and promote transparency and trust, are necessary so that communities historically excluded from the sciences can be in control of research priorities and have greater ownership of results and dissemination. We have outlined a community framework and model processes in which we share barriers, tools, and strategies for achieving successful and equitable collaborations between scientific institutions and CBOs. Finally, we challenge researchers from dominant culture institutions to self-reflect on their privilege and positionality, carefully examine a painful history of oppressive and extractive practices in the sciences, and promote more equitable and just exchange of expertise, setting the foundation for change.

## Land acknowledgment by ICBO member Karen Kitchen

We acknowledge the powerful legacy of this land's original peoples. We recognize that this country was built on Indigenous land and so we pay tribute to the hundreds of First American Nations who have stewarded these lands, these waters, and these animals since time immemorial.

We ask our First American Nations for forgiveness for the many systems that caused genocide, displacement, and exclusion. We acknowledge the great sacrifices they made in the building of our country. We want our work to repair and heal relationships between these communities and institutions.

We give thanks for the generosity of the First American Nations and celebrate their many important cultural, economic, and scientific contributions to our communities. We look to their rich ancestral knowledge to help us heal the Earth and to guide us to a deeper and respectful understanding of relationship and the interconnectedness of the human and nonhuman world. We understand that these communities sustain their sense of belonging to ancestral lands and these connections are protected through languages, oral traditions, songs, ceremonies, and stewardship.

We embrace inclusion and the concept of two-eyed seeing. Mi'kmak elder Albert Marshall said, “Two-eyed seeing is learning to see from one eye with the strengths of Indigenous knowledges and ways of knowing and from the other eye with the strengths of Western knowledges and ways of knowing and to use both these eyes for the benefit of all.”

Our work benefits from the gifts of multiple perspectives and different ways of knowing. We can create ethical spaces of engagement mindful of our responsibilities to the human and nonhuman world. Traditional ecological knowledge informs, supports, strengthens, and expands scientific ecological knowledge. And through true cocreation and collaboration, we can improve the ways in which science is done.

As we walk on this journey, we commit to making the time to explore authentic Native history and contemporary lifeways on the lands where we make our homes today. By deepening our knowledge and understanding of First American Nations we can help to foster reconciliation, cultural revitalization, and healing.

## Supplementary Material

biac001_Supplemental_File

## Author Biographical

María Cecilia Alvarez Ricalde and Juan Flores Valadez are affiliated with the Green Jay Bird Conservancy, in Cancún, Quintana Roo, Mexico. Catherine Crum, MSW is located in Washington, DC, in the United States. John Annoni is affiliated with Camp Compass Academy, in Allentown, Pennsylvania, in the United States. Rick Bonney, Mateo Luna Castelli, Marilú López Fretts, Brigid Lucey, and Karen Purcell are affiliated with the Cornell Lab of Ornithology, at Cornell University, in Ithaca, New York, in the United States. J. Marcelo Bonta is affiliated with J.E.D.I. Heart, in Portland, Oregon, in the United States. Patricia B. Campbell is affiliated with Campbell-Kibler Associates, in Groton, Massachusetts, in the United States. Makeda Cheatom and Berenice Rodriguez are affiliated with WorldBeat Cultural Center, in San Diego, California, in the United States. Yao Augustine Foli is affiliated with Ndor Eco Village, in Hohoe, Ghana. José González is affiliated with The Avarna Group, in Bozeman, Montana, in the United States. José Miguel Hernández Hurtado is affiliated with La Joven Guardia del Teatro Latino, in Syracuse, New York, in the United States. Sister Sharon Horace is located in Harlingen, Texas, in the United States. Karen Kitchen is located in Portland, Oregon, in the United States. Our dear ICBO member Pepe Marcos-Iga passed away in September 2018. Tanya Schuh is affiliated with Comunidades Latinas Unidas en Servicio, in Saint Paul, Minnesota, in the United States. Phyllis Edwards Turner and Bobby Wilson are affiliated with Metro Atlanta Urban Farm, in College Park, Georgia, in the United States. Fanny Villarreal is affiliated with Nosotros Radio, in Syracuse, New York, in the United States.
