# Supplementary material for: Understanding the Impact of Equitable Collaborations between Science Institutions and Community-Based Organizations: Improving Science through Community-Led Research
Source: Bioscience. 2022 Mar 22;72(6):585–600. doi: 10.1093/biosci/biac001 (PMC9169893; doi:10.1093/biosci/biac001)
Supplement: biac001_Supplemental_File [file biac001_supplemental_file.zip › Appendix_A-Qualitative_Survey_Interview_Protocol.docx]

**Appendix A**

**Message to the people who responded to the invitation and agreed to participate in the survey.**

Dear ________,

Thank you so much for agreeing to take this Community Perspectives survey led by representatives from Community Based Organizations throughout the country. Your answers will allow us to better understand Community Based Organization perspectives in implementing informal science education projects in under-served communities.

Please take your time answering each question. The survey is long (30 questions) and will require at least a couple of hours to answer thoughtfully. Think of it as if you were writing a chapter of a book or a blog (don’t worry about the style – just the content).

Complete the survey a little at a time so that you don’t get burned out. Just remember that if you step away from the survey you should use the same device (computer or phone) to continue.

Your participation is completely voluntary. If you feel uncomfortable at any time, feel free stop the survey without any negative consequences.

Thank you so much for everything!

The ICBO Team

**Survey/interview questions**

Your feedback in this survey will allow us to better understand Community Based Organization perspectives in implementing informal science education/citizen science projects in under-served communities. We want to learn about how partnerships with Informal Science Institutions influence implementation of projects and ways partners work together to make decisions as they plan and implement informal science in their communities.

The questions on this survey primarily focus on your experiences with partnerships and educational programming in your community. The survey should take 2-4 hours to complete (you do not need to answer all the questions in one sitting). Answering these questions should not cause you any harm or distress. However, if you do feel uncomfortable at any time, you are free to skip questions or stop the survey without any negative consequences. You may benefit from answering these questions by feeling empowered in having your voice heard.

Payment for participation: You will receive $100 gift card for participating. However, you must complete the survey to receive payment.

Your personal information will not be directly linked to your online survey answers. We want you to know that we will keep all your information confidential. Only the research team will have access to your individual responses. In any written study findings, your name will not be associated with any specific responses and care will be taken to ensure confidentiality. You will also have the opportunity to review any written study findings and provide comments before they are distributed or published beyond the project team. All electronic data will be kept on secure servers and only the research team will have access to. The ethics board that reviewed this study may also access our records for auditing purposes.

This research is being conducted by a panel of partner Community Based Organizations located in communities throughout the United States working with the Cornell Lab of Ornithology. Rick Bonney, a faculty member at Cornell University is Principal Investigator. You may contact Rick at [rickbonney@cornell.edu](mailto:rickbonney@cornell.edu) or at 607-254-2442. If you have any questions or concerns regarding your rights as a subject in this study, you may contact the Institutional Review Board (IRB) for Human Participants at 607-255-5138 or access their website at <http://www.irb.cornell.edu>. You may also report your concerns or complaints anonymously through Ethicspoint online at www.hotline.cornell.edu or by calling toll free at 1-866-293-3077. Ethicspoint is an independent organization that serves as a liaison between the University and the person bringing the complaint so that anonymity can be ensured.

Knowing this information:

- I am comfortable beginning the survey
- I do not feel comfortable filling out this survey at this time

**Your Community Based Organization (CBO) is embedded in the community you serve. Let's think about commitment, especially as it relates to collaborations or organizational partnerships with Informal Science Education (ISE) Institutions and with other CBOs in your community. (An ISE might be a large museum, zoo, or university.)**

1. Thinking about organizational partnerships or collaborations, how do you define commitment?

2. What actions demonstrate commitment in partnerships and collaborations? What are ways you know that commitment is equally shared between organizational partners?

3. How important is commitment when you get involved in a project?

4. How do you gain commitment from the leaders/partners within and between organizations?

5. How does the level of shared commitment to working in diverse communities affect programming in the community? First, think about your own CBO when answering this. Then, think about working with partner organizations. Give examples.

6. What are ways you think we can assess levels of commitment among partners?

**Now, let’s think about the realities and relevancy of being in the community. These next questions are going to ask you to think about your Community-Based Organization, and *your* experiences with this CBO in a variety of areas.**

7. Thinking broadly about your community, what are the criteria/lenses that your organization uses to identify the needs of the community? Please share with us how you assess the needs of your community.

8. If you do STEM (science, technology, engineering and mathematics) programming, how do you or your CBO gain support from the community? If you do not do STEM programming, how have you seen other CBOs gain support for it from the community?

9. What have you learned that has made STEM programs a success in your community? If you do not do STEM programming, what have you learned from others who have done it?

10. Sometimes STEM language is difficult to understand. Should we simplify the language we use in our programs and collaborations so that participants and our communities can understand the projects easily? If so, how should we do this? If not, why not?

11. How does your CBO communicate its priorities/realities to an Informal Science Education (ISE) Institution. Give examples. What are the most effective ways of doing this?

12. What can ISEs and CBOs do to be more inclusive and culturally relevant within any proposed or existing projects?

**Take just a moment, and think about organizational partnerships and relationships. Think about how they’re different and how they’re alike. The next few questions are going to ask you about trust and transparency in both organizational partnerships and relationships.**

13. So first, what actions do you believe build or demonstrate trust in organizational partnerships? And then, what actions do you think break trust in partnerships?

14. Now, think about building or breaking trust in relationships (among co-workers within your organization and in partner organizations). What actions do you think build or demonstrate trust in relationships? And what actions do you think break trust in relationships?

15. Share with us what you see as the conditions that allow for the development of trust between partner organizations.

16. Do you think organizational culture (an organization's shared values - how people behave and communicate in an organization) comes into play in building trust between organizations? If so, in what ways does it happen? Share some examples from your experience.

17. How will you know you’ve “achieved” trust in an organizational partnership? How will you know you've "achieved" trust in a relationship (with a co-worker or colleague in a partner organization)?

**The next few questions focus on transparency. We know that transparency is important between partners, so we’re going to ask you questions about the “how.”**

18. What actions do you think demonstrate transparency in organizational partnerships?

19. What seem to be the EQUALIZERS in successful partnerships? Name and define them from your experience.

20. And finally, regarding trust and transparency, there is often negotiation required between partners. When building partnerships, what are you willing to negotiate about and what not? What is flexible and what is central to your values about partnerships?

**This next set of questions asks you to consider the complex issue of Power and Privilege.**

21. What, for you, are the key drivers in partnering effectively with institutions with power (institutions that are larger, better funded, and often better positioned within the power structure of society)?

22. What do you see as the benefits for a Community Based Organization (CBO) to partner with an Informal Science Institution (ISE)? And what do you see as the benefits for an ISE to partner with a CBO?

23. What are ways you think ISEs could strengthen their local CBOs and communities? Does it matter? If so, why?

24. Funding is often a challenge for Community Based Organizations. In thinking about partnerships, what are tools or strategies that might ensure that CBOs are equitably funded or paid for their expertise? (How can you make sure that you are fairly funded for your expertise?)

25. The phrase “institutional racism” is widely used, but not necessarily with a shared definition. What is your understanding of institutional racism? Are there examples of what you see as institutional racism from your experience that you can share?

26. Do you, personally, address or want to address institutional racism in your work? How is addressing (or not addressing) institutional racism providing opportunities or barriers to successful work for you?

27. What is your individual capacity, knowledge, and skill as an individual to understand and address institutional racism?

28. Now, think about the same question related to your organization. What is your capacity as an organization to understand and address institutional racism?

29. Another important phrase is “white privilege.” What is your understanding of white privilege? Share, if you can, examples of how white privilege (or other privilege) has played out in your work.

30. You are part of this study because you have had great successes in your work and we'd like to share them with others. Please share successes in your partnerships with concrete examples. Define the actions you took that you think had a positive effect on the successful outcomes.

Is there anything else you'd like to add that you were not able to convey in the survey?

Would you be willing to participate in a follow-up interview if the research team has further questions about the answers in your survey?

- Yes
- No

We thank you for your time spent taking this survey.

Your response has been recorded.
