# Supplementary material for: Understanding the Impact of Equitable Collaborations between Science Institutions and Community-Based Organizations: Improving Science through Community-Led Research
Source: Bioscience. 2022 Mar 22;72(6):585–600. doi: 10.1093/biosci/biac001 (PMC9169893; doi:10.1093/biosci/biac001)
Supplement: biac001_Supplemental_File [file biac001_supplemental_file.zip › Appendix_B-Code-Book.docx]

**Code Book**

National Science Foundation AISL award (DRL #1422022): Collaborative Research: Examining Contextual Factors that Influence the Implementation of Projects Designed to Improve Cultural Diversity in Informal STEM Programming.

ISE—Informal Science Institution

SI—Science Institution

CBO—Community-based Organization

**Codes**

(In Vivo codes are in quotes)

ACCESS

When informal science institutions or individuals with more power use their power and connections to provide their partner organizations more direct access to funding streams, networks, and/or resources.

“ACKNOWLEDGE HISTORY”

When history of past injustices (including abuse, use, exclusion, extraction, and usurping knowledge) in the field or in previous institutional collaborations is acknowledged. Note if there is mention or intent to address/rectify past history.

ALIGNMENT

When there is alignment in mission, goals or values. Note if there is alignment in understandings of key terms like power, privilege, and institutional racism.

ARROGANCE

Self-importance. Attitude or belief in one’s superiority.

AUTHENTICITY

When authenticity is mentioned or referred to in language, actions, and when dealing with conflicts.

BENEFIT

Code when respondents mention benefit. Note if benefit is for the community or the institution.

“BIRDS OF A FEATHER”

When ISEs and CBOs prefer to work with organizations and people who look like/act like them. Similarity-Attraction Bias.

CHALLENGES

Tensions, difficulties, barriers, hurdles, mistakes in the collaboration or in relationships.

→ WORKING THROUGH CHALLENGES: Note if partners work through difficulties

COLLABORATION

When the institution and CBOs work together to implement programming, research, or achieve a goal.

COMMITMENT

Being dedicated to a goal, process, cause, idea, group, community, collaboration, project, or work.

CO-CREATION

Co-creation or equitable exchange of ideas in the collaborative and research journey and includes joint planning and goal setting, budgeting, and developing clear measures of success. Note if there is mention of co-creation before co-writing a grant proposal. Note if there is leadership involvement. Note if there is clarity on non-negotiables.

CONNECTION

Relationship or link between people.

COMMUNITY

People with shared experiences, sense of history, and belonging. In this project we are talking about communities historically excluded from the sciences. This does not include ‘community’ that is found inside institutions who hold power.

COMMUNICATION

Imparting or sharing information.

CONFLICT

When respondents mention conflict, disagreement, argument, or incompatibility between individuals or institutions

→ BRIDGE TO CONFLICT: When respondents build bridges or find commonalities or attempt to build trust when there is conflict.

CONTINUITY

When a project or collaboration continues even beyond funding. Collaboration may lead to another one that is related. Uninterrupted connection, succession, project, or collaboration.

CREATIVITY

When respondents indicate imaginative, ‘out of the box’, and/or original ideas. Note how it impacts collaborations, conflicts, research and programming.

CREDIBILITY

Who is believed, respected, or heard in the collaboration.

CREDIT

Recognition for work/authorship. Public acknowledgment.

CULTURE

Customs/attitudes/characteristics found in the community, project, and/or organizational/institutional setting.

“DO YOUR RESEARCH”

Doing research to learn about a community, organization, institution, or situation; including barriers, strengths, and realities.

“EGGSHELLS”

When respondents note that community-based organizations cannot be honest with institutional partners when they see racism, inequity, injustice, or institutional racism. CBOs feel like they are “walking on eggshells,” because addressing inequity might harm their organization or community, prevent them from getting funding, or they may be excluded from a collaboration.

“ELEPHANT IN THE ROOM”

When institutional racism, inequity, imbalance of power, or racism is present in collaborations, discussions, meetings, or relationships and it is ignored.

EQUITY/INEQUITY

When historically underrepresented populations have equal/unequal access to opportunities and networks that are capable of closing the demographic disparities. Promoting justice, impartiality and fairness within the procedures, processes, and distribution of resources by institutions or systems.

EVALUATION

Mention of formal or informal evaluation and evaluation tools, including community meetings, surveys, emergency service data, census statistics, observational research, summits, interviews, school and health data, community reports, and door-to-door canvassing.

EXCLUSION/INCLUSION

When respondents mention exclusion/inclusion at any point in the collaboration process during the planning, implementation, dissemination, and evaluation. Exclusion can take many forms, including use of technical language, a particular way of dressing, meeting venues, presence or absence of institutional staff and leadership, attention, academic degrees, dissemination channels, cultural norms, sense of belonging, sense of urgency, among others. Exclusion occurs when access to resources, power, and opportunity is limited. Note if inclusion occurs throughout the planning, implementation, dissemination, and evaluation phases. Note if inclusion is a long-term commitment—even when not funded.

EXPERTISE
Skill, knowledge, know-how.

EXPOSURE
When a CBO or ISE receives publicity/ media attention/coverage due to the collaboration.

“FACE TIME”

Code when respondents mention physical presence. Note when respondents mention that staff and leadership from the science-serving institution spend time in the community (above and beyond programming). Sharing meals together, being present at community events, volunteering or helping out, and presence at meetings. Note when leaders spend time in communities they are trying to reach.

FEAR

Emotion caused by belief that someone or something is likely to harm or threaten.

“GATEKEEPER”

When there is gatekeeping – when access to the field of science, institution, research, network, or resource is controlled, limited or only available through an institution or via institutional staff or individual. Also code when CBOs are gatekeepers to their communities.

GRANTS AND FUNDING

Code when grants or funding or money related to grants or funding streams are mentioned.

GRATITUDE AND RECOGNITION

Being thankful and appreciative. Note if it is public.

“GUINEA PIGS”

Note when respondents mention that community members feel like they are being used in research collaborations. When institutions or more resourced institutions are ‘using’ underserved communities to obtain grants, do research, and ‘check’ off diversity and inclusion requirements. Note if there is follow-through or sharing research results, and long-term commitment.

“HAVE MY BACK”

Code when STEM institutions or institutional staff use their power and/or privilege to uplift community organizations, support collaborations when funding runs out, or go out of their way to stand by their partners during difficult times.

HONESTY

Integrity, fairness, sincerity.

INEQUITABLE PAY AND CREDIT

Code when community expertise is not valued, fairly compensated, or given equitable credit in collaborations with science institutions.

INSTITUTIONAL RACISM

A systemic pattern of racism that has become established and accepted as normal behavior or part of the tradition within a society or institutions. Systematic policies, laws, practices, and distribution of resources, power and opportunities in our society/institutions that provide more benefit and access to people who are white and the exclusion of people of color.

Note if understandings of institutional racism vary depending on if you are a community organization or a more resourced institution.

INTENTIONAL

When individuals or organizations take steps on purpose or deliberately to achieve greater equity.

KNOW YOUR WORTH

When CBOs understand their power and worth and communicate it clearly when partnering with ISEs.

LANGUAGE
Expression of communication; Note if it is understandable language/lingo/technical or academic language.

LEADERSHIP
Mention of formal leadership positions or groups.

→ “LEADERSHIP BUY-IN”: Code when respondents share examples of leadership buy- in/commitment or lack of buy-in/commitment. Note if programs/research are successful.

LEARNING
Acquisition of knowledge or skills due to experience.

“LOGISTICS”

Code when respondents share details that make or break a collaboration. For instance, note when meetings, programming and communication becomes challenging when the realities of staff and communities aren’t taken into account.

MISSION

When ISEs explicitly state that collaborating equitably communities is a priority in their mission. Identifies the aim of any institution, individual, community, organization, or project.

→“LIVE THE MISSION”: When ISEs and CBOs explicitly state that working with and benefiting underserved communities is a priority in their mission and they “*live the mission*.” Institutions “live the mission” by rewarding staff and programs that directly benefit communities, ensure continuity of programming, engage leadership, include community members in all phases of co-creation and strategic planning, and have a board that reflects the community.

MOTIVATION

Code when motivation for forming collaborations and doing research or outreach in underserved communities is mentioned.

→ QUESTIONABLE MOTIVATION: When respondents question the motivation behind partnerships with ISEs. Note if respondents believe motivation is influenced by money or funding requirements, individuals’ career advancement, or other.

POWER

The ability to decide who will have access. The ability to hold, influence, or shape decision-making in research and programming. To be credible. Person or institution that holds authority or influence in a collaboration. Power to access and manage funding or networks. The capacity to influence behaviors and course of events.

PRIVILEGE

Access to benefits, resources, credibility, influence, or power only readily available to some people as a result of their advantaged social group membership. Benefit enjoyed by an individual or group beyond what is available to others.

RACISM

Prejudice, discrimination, hatred, or antagonism directed against an individual or group of a different race or ethnicity based on the belief that one's own race is superior. Can be conscious or subconscious.

REALITIES

Code when respondents mention community realities and how these play out in the collaboration. Note how community realities: language, time, money, etc… impact the collaboration. What life is really like - the world as it actually exists.

RELEVANCE

When programming, science, research, collaborations, or materials are relevant to the priorities, realities, and interests of the community.

RESPECT

Esteem, regard, or admiration.

“ROBIN HOOD APPROACH” OR “FOLLOW THE FUNDING”

When CBOs knowingly continue engaging with collaborations and partnerships that are untrustworthy, inequitable, frustrating, and/or lack transparency in order to obtain funds and opportunities that they feel can be channeled directly to their communities.

SAVIOR SYNDROME or “I KNOW WHAT YOU NEED”

When top-down programming is implemented in the community even when it is not relevant, wanted, or effective. Note if institutions believe their expertise and resources are best.

SCIENCE=ELITE

Science is perceived as being elite by communities.

“SHOWING UP”

When staff and leadership from the science-serving institution spend time in the community (above and beyond programming). Spending time in the community.

STATUS QUO

Direct or indirect mention of the Status Quo.

→“MAINTAINING THE STATUS QUO”

CBOs and ISEs behave in ways in which they are accustomed to behaving, reinforcing the status quo, or they keep playing or fitting into the roles that are expected of them. Science institutions behave in ways that maintain their power and privilege.

Note if CBOs behave in ways that are disadvantageous to themselves and their communities or may continue working in collaborations that are not equitable and may not ultimately benefit their organizations.

SUPPORT

Giving assistance, help.

“THIRD BEST MAN”

When ISEs send someone, who has no experience in the community, no authority within the institution, and no decision-making power to represent their institution at key planning and negotiation meetings.

“TIGHTROPE”

When ISEs hire one or two people who reflect/represent their communities to do outreach in historically underrepresented communities and these individuals have no decision-making power, may be short-term hires, and may be inexperienced in equity, diversity, and inclusion. Note if the hires have the ability to set the scope of their work or if the ISEs set a narrow scope for the work and have a predetermined approach set by the majority culture (the tightrope).

TRICKLE-DOWN ENGAGEMENT

When institutions put the majority of their funding, staff, and power into programming for dominant culture audiences and expect that it will “trickle-down” to the community. When SIs do “just enough” outreach to obtain funding.

TRANSPARENCY

When processes, budgets, agendas, motives, benefits, results and information are open and available to all for review and decision-making is inclusive at all times. Openness such that communities can see (easily) what is happening in the collaboration. Openness in communication, budgets, decision-making, accountability, goals, research process, benefit, motivation, etc… Transparency is closely related to power and balance of power. Note if respondents mention that transparency means different things to an SIs than it does to a CBO.

TRUST

Firm belief in the reliability, truth, ability, or strength of someone or something. Note if there is long-term commitment to the collaboration.

WALKING AWAY

Code when respondents mention leaving collaborations. Note when leaving because of inequitable collaborations.
